# Supplementary material for: Unraveling regulatory feedback mechanisms in adult neurogenesis through mathematical modelling
Source: NPJ Syst Biol Appl. 2025 Jul 26;11:82. doi: 10.1038/s41540-025-00563-5 (PMC12297322; doi:10.1038/s41540-025-00563-5)
Supplement: Supplementary file 1 — Supplementary information [file 41540_2025_563_MOESM1_ESM.pdf]

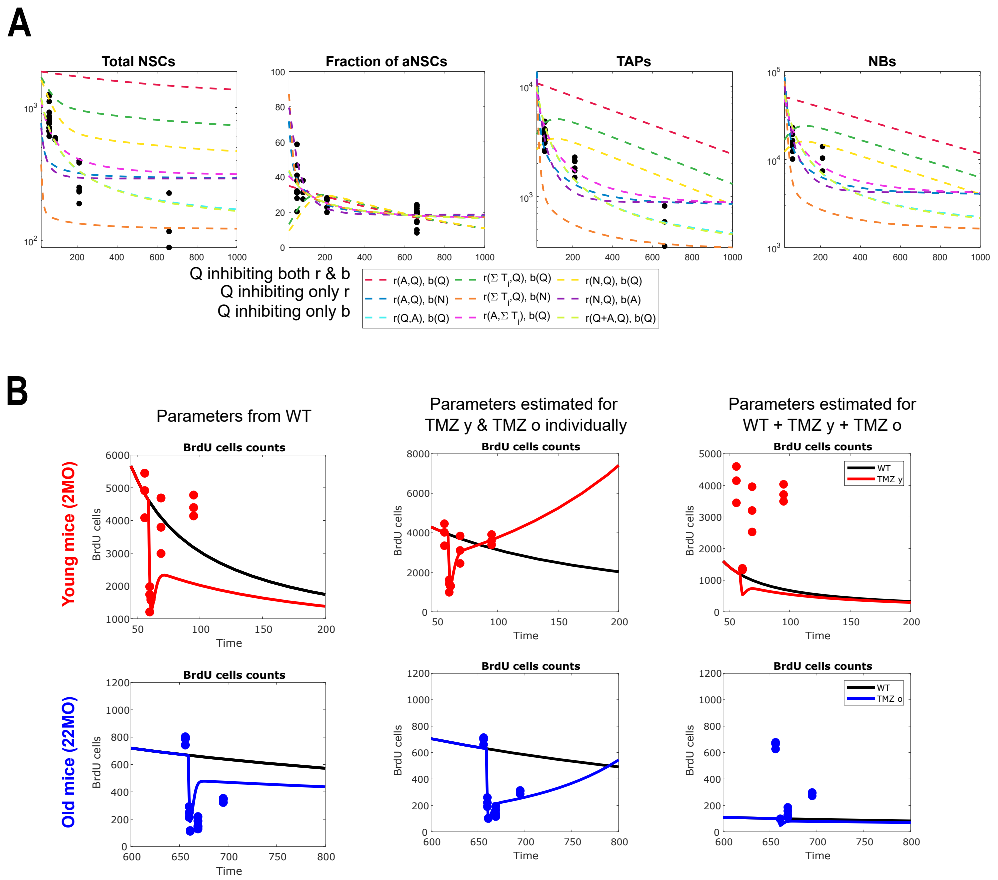

Supplementary Figure 1: Examples of bad fits to WT and TMZ data: **A.** Scenarios in which either Q inhibits both r and b, only r or only b. The estimated $b_0 > 1/2$ for all these, so the solutions converge to the positive steady state. **B.** Results of *in silico* TMZ treatment with parameters estimated for WT (left); all parameters estimated individually for young and old TMZ data (middle); and parameters estimated for fitting all data together (from WT, TMZ young and TMZ old mice). Red represents TMZ-treated young mice, blue corresponds to TMZ-treated old mice and black to WT mice without treatment. The scenario shown is that with system parameters $r(Q, A)$ and $b(A)$ (3), but the same behaviour is seen for all of the five best-scoring hypotheses (3)-(7).

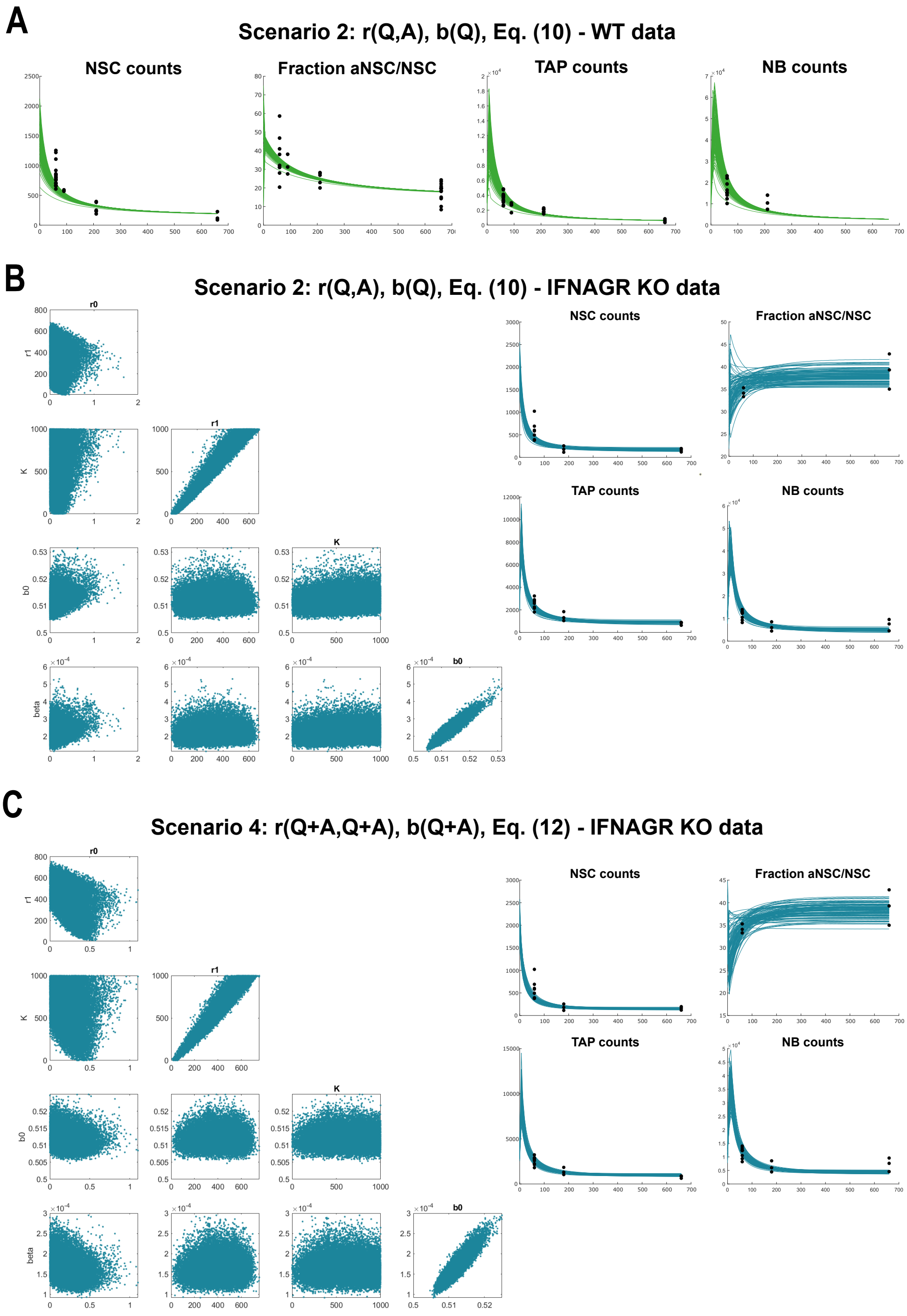

Supplementary Figure 2: Uncertainty and sensitivity quantification: **A.** Model simulations for Scenario 2 (Eq. (4)), with parameters estimated for WT data using the least-squares method, shown in Table S1, starting from a Gaussian distribution of initial conditions with mean and variance interpolated from the data (see Methods for details). All simulations quickly converge to the same dynamics, regardless of the starting values. **B.** Posterior distributions and correlations for model parameters, for Scenario 2, $r(Q, A)$, $b(Q)$, given by Eq. (10), applied to data from IFNAGR KO mice. Right-hand side inset shows the model trajectories starting from numerous initial conditions and with parameter estimates from the MCMC results (see Methods for details). **C.** Similar posterior distribution and treajectories plots as in **B.**, for Scenario 4: $r(Q + A, Q + A)$, $b(Q + A)$, given in Eq. (12).

Supplementary Table 1: Parameter values for the six scenarios

Scenario	r0_wt	K_wt	b0_wt	beta_wt	r0_ifnko	r1_ifnko	K_ifnko	b0_ifnko	beta_ifnko	pT_ifnko	pT_TMZ_y	d_TMZ_y	r0_TMZ_o	pT_TMZ_o	d_TMZ_o	rho_TMZ
r(Q,A), b(A)	2.3583	1774.3	0.49994	0.00011306	221.03	0	301.94	0.51611	0.0005284	1.052	0.44291	0.66398	0.22357	0.18146	0.96003	39.128
r(Q,A), b(Q)	2.4376	1808.5	0.50557	9.0097E-05	191.17	0.0030268	253.77	0.51106	0.0002341	1.0313	0.44968	0.71346	0.22986	0.18068	0.95958	38.744
r(Q,A), b(N)	2.2707	1735.5	0.49952	1.195E-06	191.91	0.019638	256.84	0.51306	5.4708E-06	1.1016	0.45263	0.67971	0.21505	0.18101	0.95967	39.985
r(Q+A), b(Q+A)	1.5015	1252.3	0.50355	5.0994E-05	315.52	0.21125	415.88	0.51317	0.00016873	1.0353	0.43631	0.66279	0.16984	0.18093	0.96058	40.516
r(Q,T0), b(N)	2.4994	1857.9	0.49952	1.2342E-06	227.09	0	316.7	0.51284	5.4203E-06	1.0502	0.44646	0.68597	0.23758	0.18032	0.95958	38.926
r(Q,Q+A), b(Q)	5.5205	3966.1	0.50542	8.8927e-05	521.13	0.077494	712.5	0.51192	0.00025174	1.042	0.45268	0.71795	0.52731	0.18069	0.95946	38.63

Supplementary Table 2: Akaike scores for each scenario

Scenario	AICc	∆AICc	Akaike weights
**r(Q,A), b(A)**	-1427.537237	0.749237896	23.0871787
**r(Q,A), b(Q)**	-1428.286475	0	33.57884908
**r(Q,A), b(N)**	-1424.658985	3.627490398	5.474774993
**r(Q+A), b(Q+A)**	-1428.148514	0.137960875	31.34064882
**r(Q,T0), b(N)**	-1425.007986	3.278489326	6.518548405

Supplementary Table 3: Akaike scores for the Delta-Notch and two “best” scenarios for WT data

Scenario	AICc	∆AICc	Akaike weights
**r(Q,A), b(Q)**	-728.49	0.016838	34.036 %
**r(Q+A), b(Q+A)**	-728.34	0.16275	31.641 %
**r(Q,Q+A), b(Q)**	-728.51	0	34.323 %
